# Supplementary material for: Comparative genomics provides new insights into the diversity, physiology, and sexuality of the only industrially exploited tremellomycete: Phaffia rhodozyma
Source: BMC Genomics. 2016 Nov 9;17:901. doi: 10.1186/s12864-016-3244-7 (PMC5103461; doi:10.1186/s12864-016-3244-7)
Supplement: Additional file 6: — List of orphan genes with links to PFAM (related to Additional file 1: Table S1). (ZIP 1428 kb) [file 12864_2016_3244_MOESM6_ESM.zip › BLAST_HTML_FTR/G02143_P.html]

BLAST Search Results


```
BLASTP 2.2.27+


Reference:
Stephen F. Altschul, Thomas L. Madden, Alejandro A. Schäffer,
Jinghui Zhang, Zheng Zhang, Webb Miller, and David J. Lipman (1997),
"Gapped BLAST and PSI-BLAST: a new generation of protein database
search programs", Nucleic Acids Res. 25:3389-3402.


Reference for
composition-based statistics:
Alejandro A. Schäffer, L. Aravind, Thomas L. Madden, Sergei
Shavirin, John L. Spouge, Yuri I. Wolf, Eugene V. Koonin, and
Stephen F. Altschul (2001), "Improving the accuracy of PSI-BLAST
protein database searches with composition-based statistics and
other refinements", Nucleic Acids Res. 29:2994-3005.


Database: nr
           71,551,133 sequences; 26,053,659,533 total letters


Query= G02143_P

Length=429
                                                                      Score     E
Sequences producing significant alignments:                          (Bits)  Value

emb|CED85294.1|  hypothetical protein [Xanthophyllomyces dendrorh...   837    0.0  
gb|KNB44420.1|  hypothetical protein JH06_1699 [Blastocystis sp. ...  50.1    0.004
ref|XP_011528930.1|  PREDICTED: uncharacterized protein LOC101927...  45.1    0.16 


 >emb|CED85294.1| hypothetical protein [Xanthophyllomyces dendrorhous]
Length=428

 Score =  837 bits (2163),  Expect = 0.0, Method: Compositional matrix adjust.
 Identities = 427/428 (99%), Positives = 427/428 (99%), Gaps = 0/428 (0%)

Query  1    MSNSFAGSIISSILPTDRPTRPRRSVSSLSIQFQHLEQMGSVDTSDQEQVNHAEGIASPR  60
            MSNSFAGSIISSILPTDRPTRPRRSVSSLSIQFQHLEQMGSVDTSDQEQVNHAEGIASPR
Sbjct  1    MSNSFAGSIISSILPTDRPTRPRRSVSSLSIQFQHLEQMGSVDTSDQEQVNHAEGIASPR  60

Query  61   SPTLTIDPKPLLDSKSSKKGWAFPSLAVPTQLSPTTSSSSTRPSDTRLPPTPLASKHAKP  120
            SPTLTIDPKPLLDSKSSKKGWAFPSLAVPTQLSPTTSSSSTRPSDTRLPPTPLASKHAKP
Sbjct  61   SPTLTIDPKPLLDSKSSKKGWAFPSLAVPTQLSPTTSSSSTRPSDTRLPPTPLASKHAKP  120

Query  121  TPSKLSRPLPIALSLVSSPLKGDDRPRRKKPRISSLLGSPSGSERLTTPSRVVPPSFLSA  180
            TPSKLSRPLPIALSLVSSPLKGDDRPRRKKPRISSLLGSPSGSERLTTPSRVVPPSFLSA
Sbjct  121  TPSKLSRPLPIALSLVSSPLKGDDRPRRKKPRISSLLGSPSGSERLTTPSRVVPPSFLSA  180

Query  181  SSRSRNKQSLFTHPSPSAHVSLTNPSTGFVETSSNITPEINSSPFSLAHHKSKRRSTQDQ  240
            SSRSRNKQSLFTHPSPSAHVSLTNPSTGFVETSSNITPEINSSPFSLAHHKSKRRSTQDQ
Sbjct  181  SSRSRNKQSLFTHPSPSAHVSLTNPSTGFVETSSNITPEINSSPFSLAHHKSKRRSTQDQ  240

Query  241  VGSRSLMSGLMGPGLLADQAGSKELSVRDVLLGKSPTKGKIAHGLLFSNASLLLSSHQTS  300
            VGSRSLMSGLMGPGLLADQAGSKELSVRDVLLGKSPTKGKIAHGLLFSNASLLLSSHQTS
Sbjct  241  VGSRSLMSGLMGPGLLADQAGSKELSVRDVLLGKSPTKGKIAHGLLFSNASLLLSSHQTS  300

Query  301  LSKFYTALHKTTLSNTSSPLKPDINLTVRRVIPSSLSPRRMILETCMSTSILAESTEDQI  360
            LSKFYTALHKTTLSNTSSPLKPDINLTVRRVIPSSLSPRRMILETCMSTSILAESTEDQI
Sbjct  301  LSKFYTALHKTTLSNTSSPLKPDINLTVRRVIPSSLSPRRMILETCMSTSILAESTEDQI  360

Query  361  RWVILDETAQENAVPKNISNWSALKEGSTVLGFRPFREIEWDGLGALIREQRMDLNKKIL  420
            RWVILDETAQENAVPKNISN SALKEGSTVLGFRPFREIEWDGLGALIREQRMDLNKKIL
Sbjct  361  RWVILDETAQENAVPKNISNRSALKEGSTVLGFRPFREIEWDGLGALIREQRMDLNKKIL  420

Query  421  LIDKFLIT  428
            LIDKFLIT
Sbjct  421  LIDKFLIT  428


>gb|KNB44420.1| hypothetical protein JH06_1699 [Blastocystis sp. ST4]
Length=1446

 Score = 50.1 bits (118),  Expect = 0.004, Method: Composition-based stats.
 Identities = 62/258 (24%), Positives = 99/258 (38%), Gaps = 39/258 (15%)

Query  1    MSNSFAGSIISSILPTDRPTRPRRSVSSLSIQFQHLEQMGSVDTSDQEQVNHAEGIASPR  60
            + NS    I   I P   P  P   V S     Q  ++   + T+ ++ +  +  + SP 
Sbjct  480  VDNSTVPQIAKDISP---PPFPDVQVPS---TVQQSDKTHDISTAMKDSIKISSELMSPS  533

Query  61   SPTLTIDPKPL-----LDSKSSKKGWAFPSLAVPTQLSPTTSSSSTRPSDTRLPPTPLAS  115
              + T+ PK L     L           P L   + + P    S + P+D+++ P P + 
Sbjct  534  GSSSTLPPKSLPADIHLSPFPQPSQPVLPPLRTDSHILPFPQPSQSLPTDSQVSPLPQSF  593

Query  116  KHA----KPTP-SKLSRPLPIALSLVSSPLKGDDRPRRKKPR-------------ISSLL  157
            K +     PTP S+ S P    ++  S P    D P    P+             ++  +
Sbjct  594  KSSPLNESPTPPSRQSLPSDSGVTQPSLPSLPADSPVSPFPQLCQSLPSSLPSDSVAPQI  653

Query  158  GSP-----SGSERLTT---PSRVVPPSFLSASSRSRNKQ--SLFTHPSPSAHVSLTNPST  207
             SP     S S+   +   P+   PP  LS SS + + Q    FT P P       NPS 
Sbjct  654  QSPFPTVSSVSQFFNSSAFPNNSQPPQILSPSSLNCDTQLSQSFTSPIPDELHQCENPSQ  713

Query  208  GFVETSSNITPEINSSPF  225
              +   S+++P    SPF
Sbjct  714  SLILDHSDVSPLFPQSPF  731


>ref|XP_011528930.1| PREDICTED: uncharacterized protein LOC101927859 isoform X5 [Homo 
sapiens]
Length=1974

 Score = 45.1 bits (105),  Expect = 0.16, Method: Composition-based stats.
 Identities = 70/295 (24%), Positives = 113/295 (38%), Gaps = 19/295 (6%)

Query  58    SPRSPTLTIDPKPLLDSK-----SSKKGWAFPSLAVPTQLSPTTSSSSTRPSDTRLPPT-  111
             SP + +LT  P     S+     S K+     SL      +  TS+ ST  S TR+PP  
Sbjct  1624  SPPTASLTRSPSTASLSRTPPRASLKRSPPTASLTRTPSRASLTSTPSTASSLTRMPPKQ  1683

Query  112   ------PLASKHAKPTPSKLSRPLPIALSLVSSPLKGDDRPRRKKPRISSLLGSPSGSER  165
                   P+AS    P  +  +R LP A    + P     R     P  +SL  +PS + R
Sbjct  1684  SLSRTPPMASLTRSPPTASRTRTLPRASRTRTPPRASLTR----SPPTASLRRTPSRASR  1739

Query  166   LTTPSRVVPPSFLSASSRSRN-KQSLFTHPSPSAHVSLTNPSTGFVETSSNITPEINSSP  224
               TP R         +S +R   ++L T    +A +  + P+     T S  +     S 
Sbjct  1740  TRTPPRASRTRTPPRASHTRTPSRALLTRTPSTASLKRSPPTASLTRTPSRASLTRTPSR  1799

Query  225   FSLAHHKSKRRSTQDQVGSRSLMSGLMGPGLLADQAGSKELSVRDVLLGKSPTKGKIAHG  284
              SL   KS  R++  +  SR+ ++       L     +   +       ++ T  + +H 
Sbjct  1800  ASLTRLKS--RASHTRTPSRASLTRTPPTASLTRSPPTASRTRTPPRASRTRTPPRASHT  1857

Query  285   LLFSNASLLLSSHQTSLSKFYTALHKTTLSNTSSPLKPDINLTVRRVIPSSLSPR  339
                S ASL  +    SL +       T +  T S  +     ++ R+ P +  PR
Sbjct  1858  RTPSRASLTRTPSTASLKRSPPMASLTRMPPTRSLSRTPPAASLTRLPPRASLPR  1912


Lambda      K        H        a         alpha
   0.313    0.127    0.359    0.792     4.96 

Gapped
Lambda      K        H        a         alpha    sigma
   0.267   0.0410    0.140     1.90     42.6     43.6 

Effective search space used: 4169348786784


  Database: nr
    Posted date:  Sep 23, 2015 12:05 AM
  Number of letters in database: 26,053,659,533
  Number of sequences in database:  71,551,133


Matrix: BLOSUM62
Gap Penalties: Existence: 11, Extension: 1
Neighboring words threshold: 11
Window for multiple hits: 40
```
